# High‐frequency (20 MHz) high‐intensity focused ultrasound: New Treatment of actinic keratosis, basal cell carcinoma, and Kaposi sarcoma. An open‐label exploratory study

**DOI:** 10.1111/srt.12883

**Published:** 2020-06-17

**Authors:** Jørgen Serup, Torsten Bove, Tomasz Zawada, Alexander Jessen, Mattia Poli

**Affiliations:** ^1^ Department of Dermatology Bispebjerg University Hospital Copenhagen Denmark; ^2^ TOOsonix A/S Horsholm Denmark

**Keywords:** cure rate, guidance, high‐intensity focused ultrasound, pain, photodynamic therapy, premalignant, scar, sequelae, skin cancer, verruca

## Abstract

**Background:**

Skin cancer is common, growing, challenging, and in need of progress in early‐stage treatment. 20 MHz high‐intensity focused ultrasound (HIFU) is new and applied to actinic keratosis (AK) and skin cancers for the first time. HIFU of lower frequency is already used in the treatment of internal cancers.

**Materials and Methods:**

Eight patients with 201 AK lesions, one patient with 7 basal cell carcinomas (reoccurrences after PDT), and one patient with 7 Kaposi sarcoma lesions (4 treated with radiotherapy in the past) were given 1‐3 HIFU treatments. Twenty megahertz HIFU was dosed as 150 ms at 0.6‐1.2 J/shot applied to target lesions. Probes with different target depths were available. The preferred shot energy and focal depth in AK were 0.9 J and 1.3 mm. A “Sandwich” strategy with HIFU applied in two depths were tried in cancers. The follow‐up period was 3‐6 months.

**Results:**

All AK cleared except 5, giving a cure rate of 97%. Post‐treatment lesion healed in 1‐2 weeks with no scar. VAS pain was from 1 to 8, and in any case less than experienced with previous PDT. In both basal cell carcinoma (BCC) and sarcoma, healing was confirmed by histological verification.

**Discussion/conclusion:**

20 MHz HIFU was an effective and safe treatment of AK. This new treatment, applicable to any anatomical site, has promising advantages relative to PDT and has the potential to replace or supplement PDT in future. Case‐observations indicated that HIFU can be useful in skin cancers as well.

## 1 INTRODUCTION

Non‐invasive high‐intensity focused ultrasound (HIFU) operating at frequencies from 500 kHz to approximately 3 MHz has gradually been established as an efficient non‐invasive treatment of internal cancers of major organs, bone metastases, and cerebral pathologies over the last decade.^1^, ^2^, ^3^, ^4^, ^5^, ^6^, ^7^, ^8^, ^9^ HIFU focal points are positioned deep within the body with the anatomical location guided by MRI scanning or ultrasound imaging. In the focal point, temperatures of about 65°C are achieved, which is enough to kill internal cancer cells without affecting superficial or adjacent structures.

**Figure 1:**
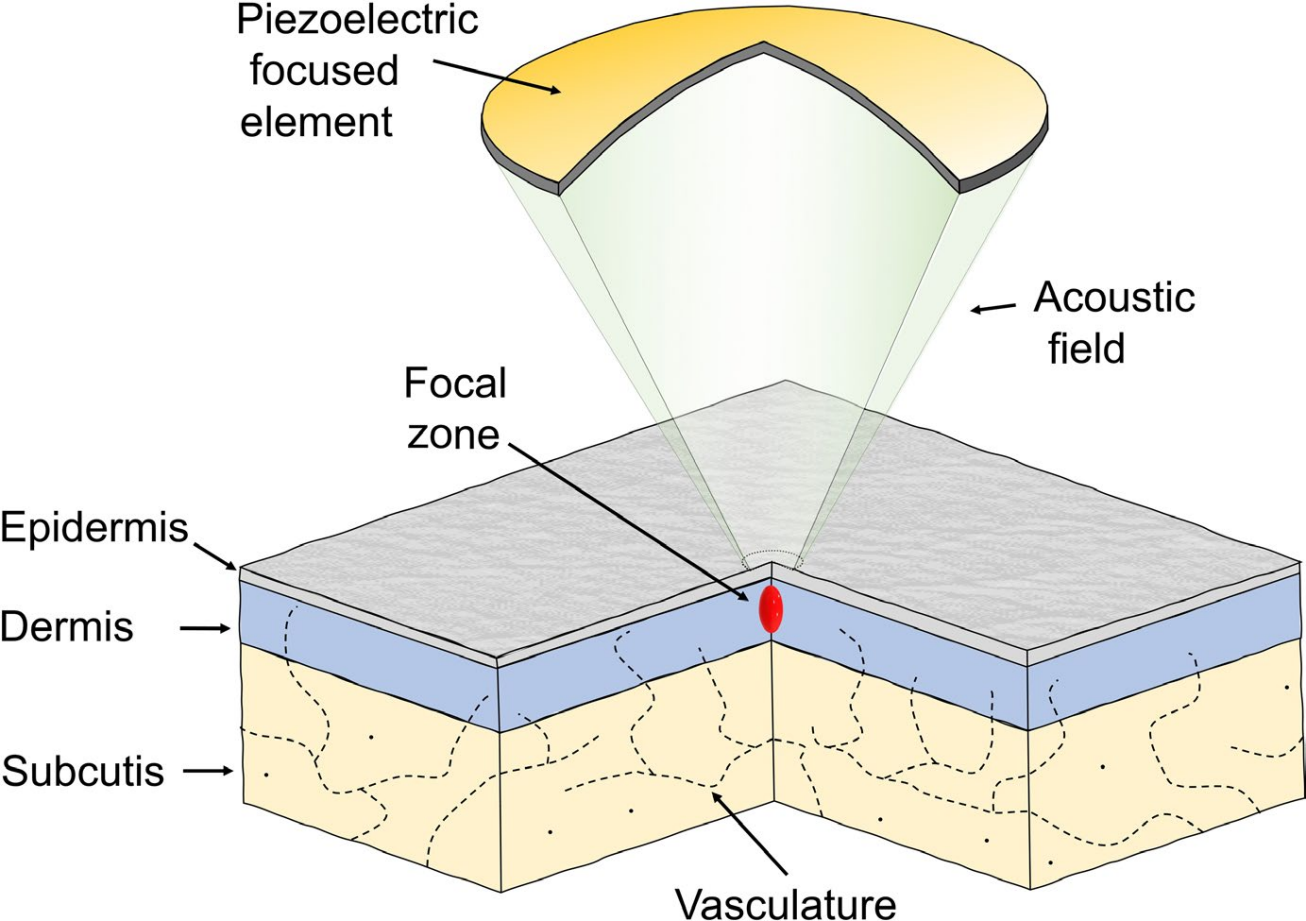
Schematic illustration of high‐intensity focused ultrasound (HIFU) and skin. The transducer is a focused piezoelectric element constructed to deliver non‐invasive acoustic energy of a high intensity into a confined focal zone below the skin surface. In the focal zone and the adjacent part of the skin above the focal point heating, the ultrasound energy is absorbed and transduced into a temperature insult. In contrast, the energy attenuates abruptly below the focal point. The increase in temperature depends on the power and duration of the ultrasound beam

It has recently been demonstrated in preclinical studies that HIFU operating at high frequency with a close focal distance can target selected small objects in media near the surface of the medium and reproducibly deliver a thermal insult.^10^, ^11^ The dose‐effect curve in the range of interest has been demonstrated to be linearly dependent on energy, and thereby allow for the accurate dose‐effect modulation needed in clinical applications. High‐frequency HIFU therefore becomes relevant to dermatology, plastic surgery, and cosmetology. The principle of HIFU is illustrated in Figure 1.

The size of a focal zone generated by a HIFU transducer is inversely dependent on the operating frequency; that is, the higher the frequency, the smaller the focal zone. For HIFU treatments to be relevant for dermatology, the focal zone needs to be positioned and confined accurately within the epidermis, dermis, or subcutis depending of the purpose of the target and the desired intervention. As demonstrated in the preclinical testing, accuracy in skin targets requires an operating frequency of approximately 20 MHz.^10^ Commercially available systems dedicated to various types of body contouring, thus primarily targeting larger volumes in subcutis or below, are however typically operating at 4‐10 MHz.^12^, ^13^ Such systems can therefore not be used for confined targets in the epidermal and dermal layers.^14^, ^15^

In the broad spectrum of dermatological diseases, premalignant lesions and skin cancers are of major interest, inspired by the above documented efficacy of HIFU on internal cancers.

Actinic keratosis (AK), basal cell carcinoma (BCC), and squamous cell carcinoma (SCC) are common, and an increasing incidence is noted along with the growing proportion of elderly people.^16^, ^17^ BCC is the most common type of cancer in man. These epidermal pathologies are straightforward and logic first indications for the novel 20 MHz HIFU used as an ablative method. Kaposi sarcoma (KS) originating in the vasculature of the dermis is another potential indication, albeit being special and rare. Malignant melanoma is at this stage not within reach of the HIFU method. Benign tumors and conditions, such as common warts, condylomas, molluscum contagiosum, and syringoma are potential future indications.

The effect of 20 MHz HIFU, as mentioned above, was recently studied in pre‐clinical laboratory studies, which included a study on Göttingen minipigs.^11^ The study on pigs was designed to document local safety and efficacy, and issues of general safety. This study also produced key information on operation and dose settings needed before human use. It was observed that HIFU produces an immediate wheal and flare reaction resulting from the thermal trauma in the focal point. Treatments furthermore have a horizontal dimension; that is, nearby treatment points applied “shoulder‐by‐shoulder” behave in synergy, and the wheal and flare reaction and ablative effect are stronger in comparison with an individual single point dosage. Treatments were generally well tolerated without persisting adverse findings at the end of the 3‐month follow‐up.

Used as an ablative method, the 20 MHz HIFU by intention produces a superficial wound that may heal over days or weeks. Scar sequelae were not observed at end of the pig study by clinical reading. Sporadic cases with fibrous change in the dermis by histology were observed. This was seemingly associated with flattening of skin surface markings, but only observed in a few cases. The 20 MHz HIFU method used in the preclinical studies was therefore concluded to be both efficient with respect to production of controlled skin ablations, and of acceptable safety, thus ready for clinical application. The method was reproducible and tunable in the dosage range relevant for skin pathologies.

The study presented below is the very first study of the novel 20 MHz HIFU method applied to premalignancies and malignancies of human skin. No guidance and no past study were available from the medical literature. A clinical material is presented, supplemented with illustrative case reports.

## 2 MATERIALS AND METHODS

### 2.1 Patient recruitment

The study was open label and integrated in the ongoing clinical treatment practiced at the Department of Dermatology of Bispebjerg University Hospital, Denmark. HIFU treatment was offered as an optional treatment to patients with AK, BCC, and rare neoplasia, when conventional treatments had failed, were limited by pain associated with treatments, or on patient's special preference. This included patients, who for different personal reasons, abstained from routine therapy. Thus, fixed standards of in‐ and exclusion criteria were not used. The material, however, was consecutive and included every treated patient. The study was an open‐label production control assessment. The study was conducted from November 2018 to February 2020. Patients were informed before treatments and gave their consent. The principles of the Helsinki Declaration II were followed.

### 2.2 Equipment

**Figure 2:**
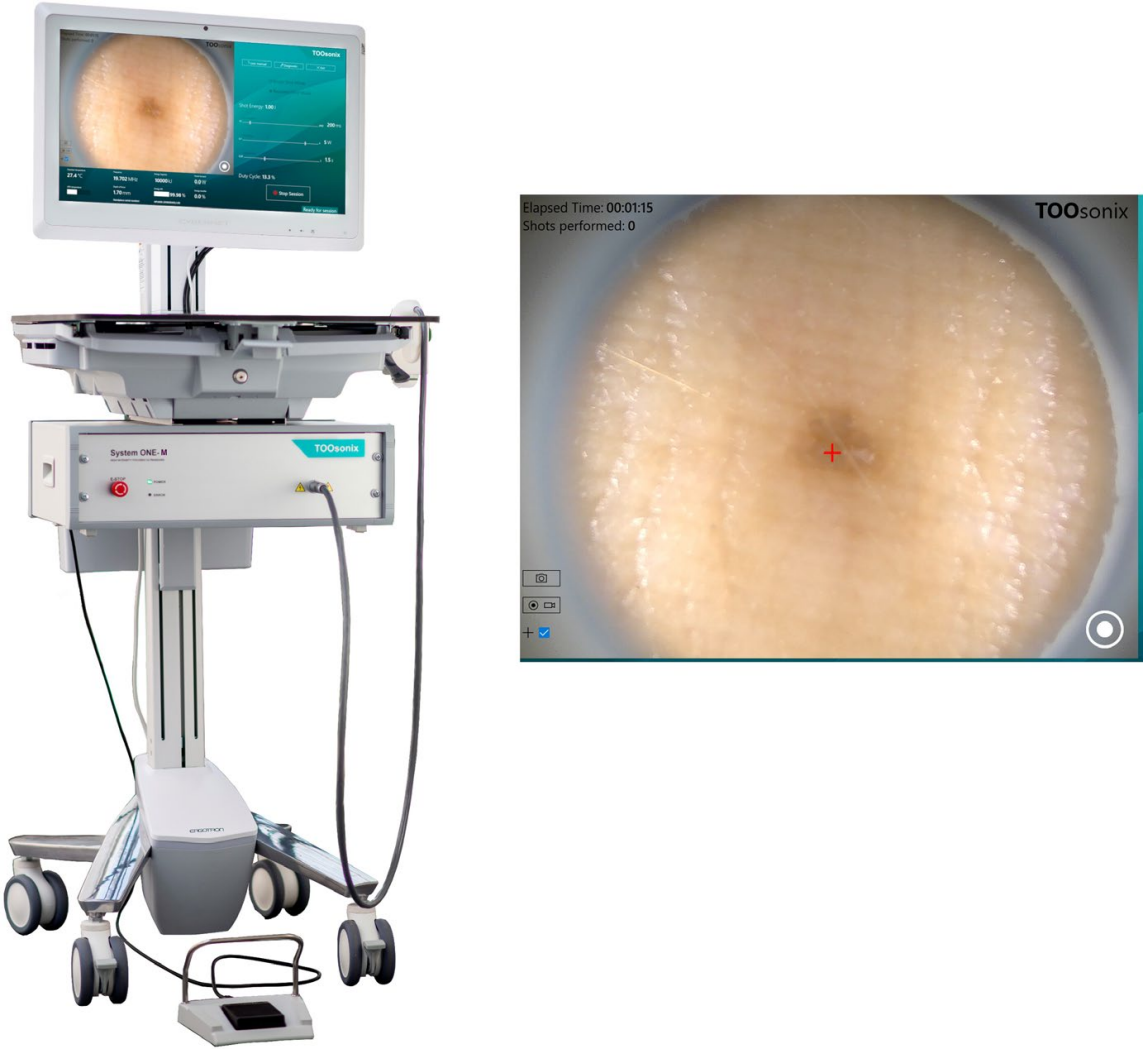
TOOsonix System ONE with a close‐up photograph of the dermascope picture of a suggested target lesion filmed real time through the probe. The red cross indicates the horizontal location of a high‐intensity focused ultrasound (HIFU) shot by activation of the foot switch

Treatments included in this study were performed using the commercially available System ONE from TOOsonix A/S, Denmark ^18^, ^19^; Figure 2. The system operates at 20 MHz ± 5%. The HIFU equipment was safety tested and approved and registered by the Dept. of Medical Engineering of the Hospital. The equipment fulfills the general requirements for basic safety and essential performance according to IEC 60601‐1:2006 including its collateral standards.

The system consists of an ultrasound power unit responsible for generation and regulation of ultrasound signals, handpieces with a range of ultrasound transducers, and a software to manage treatment settings. HIFU doses, or “shots,” are activated manually by a footswitch. High‐resolution real‐time monitoring of the treated area is integrated in the system using a digital video camera that operates as a dermascope. Different handpieces of the system are characterized by their −6 dB focal zone depth, that is, the maximum extend of the zone where acoustic intensity is within 25% of the maximum intensity in the center of the zone. Handpieces with focal depths ranging from 1.1 to 2.7 mm were available and selected depending on the indication and vertical position of the lesion to be treated.

### 2.3 HIFU start up, setting, and dosing

Prior to treatment, the transducer chamber was filled with non‐gaseous distilled water and closed with a thin polyethylene film. A standard ultrasound coupling gel was used between the skin surface and the probe.

In cases when the target lesion foreseeably would be difficult to identify with the integrated dermascope due to lack of contrast to the surrounding skin, the target lesion was marked with a pen. It was found important that the pen was water‐resistant to prevent diffusion of the stain into the water‐based gel, and thus causing blurring of the view. AKs were routinely smeared with 2.5% iodine in ethanol applied using a cotton swab. The crusted and keratotic areas in an AK absorb and bind yellow iodine, and the contrast to surrounding skin with normal skin surface ridges is thereby improved. Iodine at the same time disinfects the area of treatment.

The duration of each ultrasound shot was chosen at 150 ms in all treatments. This has previously been found adequate for sufficient energy transfer, and at the same time minimize influence of movement of the handpiece during HIFU transmission. The acoustic shot energy was preferably 0.6, 0.9, or 1.2 J/shot depending on the thickness of the target, the dept in the skin, and other characteristics such as major keratosis or scaling on the top of the lesion. Such dry and possibly air‐containing structures potentially weaken the ultrasound transmission dramatically. It may therefore be necessary to soak such lesions with ultrasound gel or some water‐saturated cotton pad some 5‐10 minutes before treatment to increase the humidity within such keratotic barriers. Curettage of loose keratotic material also may be used, followed by hydration.

Guided by a red pointer on the screen, and observing the skin surface with the integrated dermascope camera, the HIFU shot was positioned precisely over the lesion target. The system was activated with the foot switch, and a shot was fired. Whitening or contraction of the treated skin was displayed directly on the screen in real‐time and surveyed to control that an effective dose was received by the skin target. A full treatment consisted of consecutive shots administered shoulder‐by‐shoulder, that is, placed with approximately 2‐3 mm between their centers, to fully cover the targeted area. Shots were administered at intervals of approximately 1‐2 seconds.

Optimal dosing requires the probe to be held precisely perpendicular to the skin surface. A boiling sound impulse as a shot is fired indicates the angle was not right, and air bobbles in the coupling gel had absorbed the energy.

The HIFU treatment produces the intended thermal burn lesion in the target, often followed by a wheal and flare response that peaks after 5‐10 minutes and fades over the subsequent 10‐30 minutes. After a few days, a superficial dry wound may occur. The wound will heal spontaneously over a few days or weeks after a phase of inflammation and possibly crustation.

High‐intensity focused ultrasound treatment causes instant pain directly when the shot is fired. Pain was measured on a visual analogue scale, ranging from 0 to 10 with 10 marking unbearable pain.

## 3 RESULTS

In total eight patients with AK, one patient with BCC and one patient with Kaposi sarcoma participated in the study, see Table 1. Additionally, two patients with common warts had been treated.

**Table 1:** Summary of treatments in study

Subject	Diagnosis	No of lesions	Probe/Energy (Joule)	VAS Pain Median (Range)	Outcome
LV	AK	59	1.3 mm/0.6‐0.9 J	5 (2‐9)	All cleared No sequelae
AF	AK	40	1.3 mm/0.4‐0.9 J	8 (7‐9)^a^	All cleared except 3 No sequelae
EJ	AK	51	1.3 mm/0.9 J	2 (1‐3)	All cleared No sequelae
ED	AK	8	1.3 mm/0.9 J	1 (1‐2)	All cleared No sequelae
OB	AK	10	1.3 mm/0.9 J	6 (2‐9)	All cleared except 2 No sequelae
KB	AK	9	1.3 mm/0.6 J	4 (3‐6)	All cleared No sequelae
EB	AK	1	1.3 mm/0.9 J	2 (2)	All cleared No sequelae
JR	AK	23	1.3 mm/0.6‐1.2 J	4 (1‐7)	All cleared No sequelae
SA	BCC^b^	7	1.7 mm/1.2 J	5 (1‐7)	Cured Negative follow‐up histology No sequelae
BK	KS^c^	7	1.7 mm/1.2 J^d^ 1.3 mm/0.9 J^d^	3 (2‐4)	Cured Negative follow‐up histology. No scar Remaining hyper‐pigmentation (iron blood stain)

Subjects were treated with HIFU applied in one session in limited disease, and 1‐3 sessions in widespread disease, determined from number of lesions. Follow‐up ranged from 3 mo to 1 y.

Abbreviation: AK, actinic keratosis.

^a^ Subject suffered from very sensitive skin.

^b^ Recurrence after treatment with photodynamic therapy;

^c^ Recurrence or no effect after radiotherapy, or untreated.

^d^ Treatment applied as “Sandwich” treatment with deep high‐power treatment followed by shallow lower power treatment given in the same session, on the top of deep treatment.

### 3.1 Actinic keratosis

Actinic keratosis or “solar keratosis” is an in situ dysplasia resulting from sun exposure. The dysplasia evolves from the basal cells of the epidermis, and thus, it is superficial and easy to diagnose. AK is found in sun‐exposed skin particularly in the laded scalp, the face, and on the arms. It is the commonest epithelial precancerous lesion and a precursor of BCC and SSC. Efficient treatment of AK is cancer prevention. Some individuals are especially prone to AK and skin cancers and need efficient treatment.

Eight patients with AK and 201 lesions were treated with 20 MHz HIFU. The observation period was from 6 weeks to 6 months. Photographs were taken in every case to document the precise anatomical position of the AK and support follow‐up. All AK cleared except 5, and the success rate was 97%. The non‐cleared AK seemed to be those of uncertain demarcation toward healthy skin, those with a dense keratosis on the top or cases with a history of being especially recalcitrant and resistant to previous therapies. Lesions in patients with baldness and a glossy and oily skin surface were difficult to mark with a pen and with iodine solution, and the reference to surrounding skin having entirely flat skin surface relief often was uncertain. Three illustrative cases are presented below.

#### 3.1.1 Case report 1, actinic keratosis on the scalp

**Figure 3:**
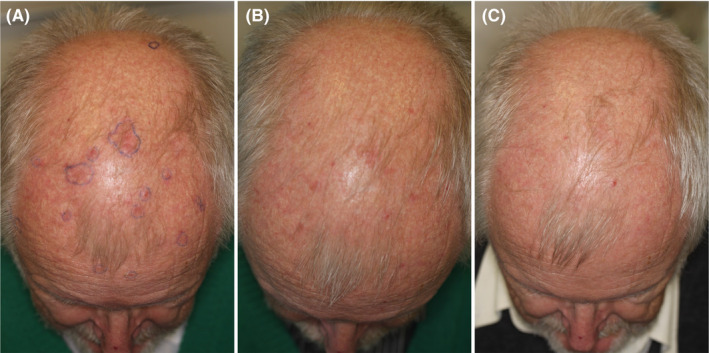
Case 1. A, before high‐intensity focused ultrasound (HIFU); B, follow‐up after 3 wk, inflammation of end‐stage healing; C, 3 mo after HIFU, AKs cleared

Subject (AF) was a 70‐year‐old male with multiple AK on the scalp, ears, and hands since several years. He had worked in Africa and been sun exposed for a long period. Subject had undergone several treatments with photodynamic therapy (PDT) and cryosurgery, but complained about treatment failures, recurrences, and bleedings (he received anticoagulation treatment). PDT had been given up due to extraordinary pain, described as far above VAS 10! Subject suffered from peripheral neuropathy making him especially sensitive to pain. Subject therefore requested HIFU. His treatment is shown in Table 1 and illustrated in Figure 3. HIFU was painful to him with median VAS 8, however, acceptable in contrast to pain previously experienced on PDT. He was given two follow‐up treatments of new lesions including AKs on trunk and extremities.

#### 3.1.2 Case report 2, actinic keratosis on the neck

**Figure 4:**
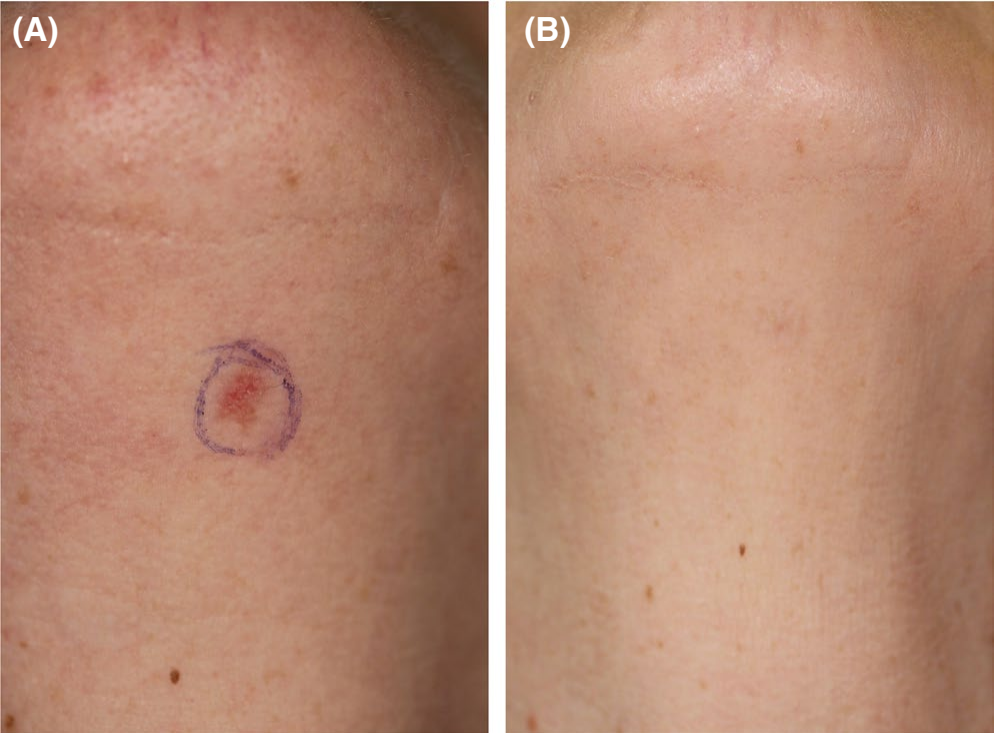
Case 2. High‐intensity focused ultrasound (HIFU) treatment of actinic keratosis (AK) on the neck. A, before HIFU treatment; B, 6 mo after HIFU. There was no scar and no dyspigmentation

Subject (LV) was a 61‐year‐old female with multiple AK on legs, hands, face, and the neck. Subject had undergone several treatments with PDT, VAS pain rated 10, and with cryosurgery over the past years, but complained about constant recurrences and treatment failures. Oral retinoid had no effect. Subject requested HIFU treatment as a potentially more effective and less painful treatment option. Her treatment is shown in Table 1 and exemplified in Figure 4. She had been given two further treatment sessions on other parts of the body. She constantly and independent of anatomical site reported pain by VAS to be less in comparison with pain experienced during PDT.

#### 3.1.3 Case report 3, actinic keratosis on the earlobe

**Figure 5:**
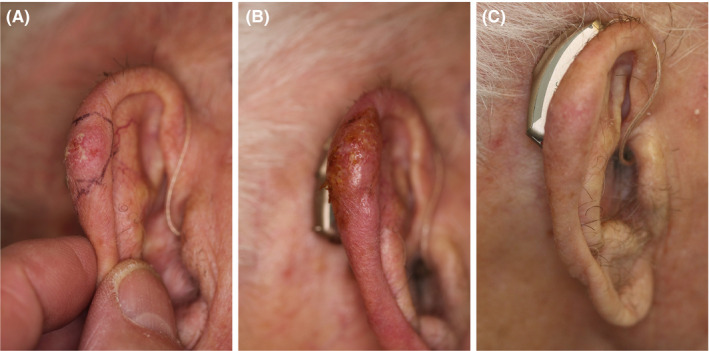
Case 3. A, before high‐intensity focused ultrasound (HIFU) treatment; B, immediately after HIFU, with treatment inducing minor wheal and flare reaction. C, 2 wk after HIFU treatment; some post‐treatment inflammation, however, without subjective complaints. At follow‐up after 3 mo, the actinic keratosis (AK) was flattened and healed, and patient felt satisfied. No recurrence as observed at a 6‐mo follow‐up visit

Subject (KB) was a 97‐year‐old male with multiple AK on the face, ears, and hands. Subject has undergone several treatments with PDT and cryosurgery but complained about many recurrences and one especially cumbersome AK on the earlobe, which disturbed his sleep. Subject requested HIFU hoping for clearing. His treatment is shown in Table 1 and illustrated in Figure 5. After 2 weeks, the AK was barely visible, and the skin had healed almost completely. Subject reported nearly no pain or tenderness, and he could sleep without problems from the treated site.

### 3.2 Basal cell carcinoma

Basal cell carcinoma is the commonest cancer in man and sun induced, sometimes originating from an AK. It originates, as the name indicates, from proliferating basal cells of the epidermis. It may be nodular, flat, or ulcerating, with invasion of the dermis horizontally and vertically. Some patients have many BCC originating de novo or seen as reoccurrences, when treatments such as PDT have failed.

#### 3.2.1 Case report 4, recurrent BCC

**Figure 6:**
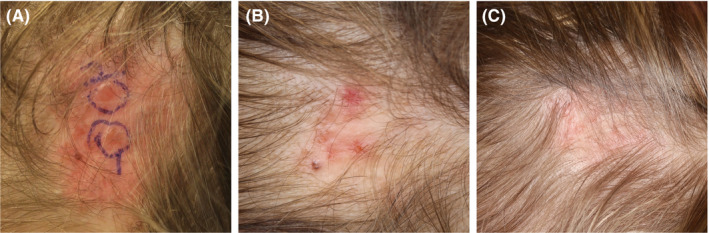
Case 4. A, before high‐intensity focused ultrasound (HIFU), presenting two larger recurrences encircled with a blue pen. B, after 2 wk; treated lesions were in late healing phase. On this occasion, 5 minor lesions were treated with HIFU. C, after 2 mo. Subsequent follow‐up after 6 mo showed no reoccurrence. Follow‐up biopsy from one initial lesion, see A, taken after 6 mo showed no basal cell carcinoma

Subject (SA) was a 67‐year‐old female with 7 recurrent BCCs confirmed by histology or clinical examination was treated with HIFU. The lesions were grouped in a 3 cm spot in the scalp. She had been treated with PDT, which initially cleared the original BCC, however, with hair loss as a side effect. She refused further PDT, surgical excision, and radiotherapy, but accepted HIFU. Initial HIFU treatment was given as 25 shots to two larger recurrences confirmed by histology (2‐mm punch biopsy) and 5 minor recurrences according to clinical diagnosis and examination with a magnifying lens. Aiming at radical therapy, the setting was more powerful and deep, that is, probe dept 1.7 mm and power 1.2 J/shot. Her treatment is shown in Table 1 and illustrated in Figure 6.

### 3.3 Kaposi sarcoma

Kaposi sarcoma is a viral multifocal tumor characterized by full‐thickness vascular proliferation of the dermis, thus, very different from BCC, the latter originating from proliferating keratinocytes of the basal cell layer of the epidermis, and mostly caused by long‐term sun exposure. Immunodeficient patients, particularly HIV patients, are predisposed. There is often leakage of erythrocytes across the pathological vascular wall into the dermis causing semi‐permanent iron oxide stain, for example, a kind of spontaneous tattoo. Radiotherapy is the treatment of choice, but not all sarcomas respond to this treatment, and new sarcomas in other loci often show up over time.

#### 3.3.1 Case report 5, Kaposi Sarcoma

**Figure 7:**
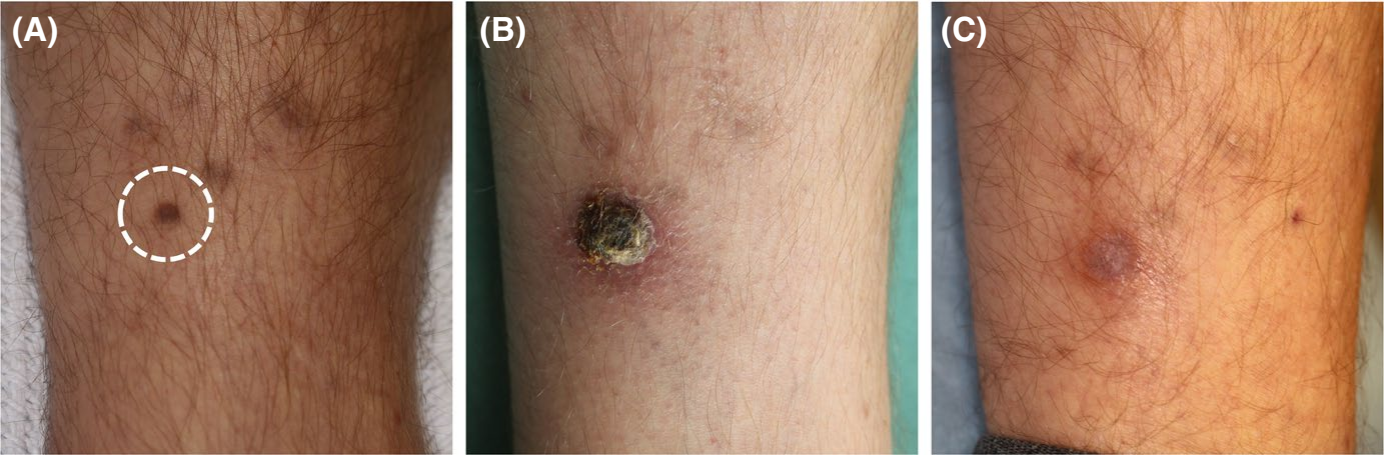
Case 5. A, Kaposi sarcoma prior to high‐intensity focused ultrasound (HIFU) treatment. B, treated site 10 wk later. A thick crust of keratotic material and debris had developed, however, easily removed. C, same after 10 mo. Histology showed no sarcoma, but the esthetic outcome was less satisfactory with remnant iron stain and some inflammation. The cosmetic result may improve over time. The illustrated lesion shown was a worst case; the other 6 lesions healed without complications and no special crustation, and some remnant iron stain

Subject (BK) was a 48‐year‐old immunodeficient male with Kaposi sarcomas on his legs, confirmed by histology. 7 sarcomas needed treatment. 4 lesions had been treated with radiotherapy in the past, but these lesions appeared not to respond, and new sarcomas had developed as separate new elements. New sarcomas regularly came up. He therefore needed some easy‐to‐practice treatment schedule. He was scanned with an ultrasound scanner (Dermascan‐C; Cortex Technology) producing cross‐sectional ultrasound images of the skin. Scanning showed echo‐poor fields with unsharp demarcation mainly in the lower dermis directly at the interface to subcutis. Thus, a deeper HIFU focus and a higher power was logic. Each sarcoma was administered a two‐level “Sandwich” treatment, that is, an initial treatment with a 1.7 mm probe and 1.2 J/shot, directly followed by a cover‐up treatment with a 1.3 mm probe and 0.9 J/shot. His treatment is shown in Table 1 and illustrated in Figure 7. Clinical follow‐up indicated no reoccurrence albeit clinical observation is hampered by the semi‐permanent iron oxide stain. However, two punch biopsies taken after 8 months showed no sign of Kaposi Sarcoma and, as expected, iron oxide deposits, and some fibrosis.

### 3.4 Variae, common warts

Two cases of verruca vulgaris, each case one wart, were treated with HIFU. The warts had been resistant to previous therapies given in the outpatient clinic. Both warts, one on a finger and another on the foot, were massive with a dense keratotic massive 1‐2 mm in thickness. There were no wheal and flare response, no inflammatory reaction, and therapeutic effect of HIFU. The cases illustrate that HIFU may be stopped by a thicker keratotic cover on a lesion. In AK‐lesions with major keratosis, it appears rational to humidify or remove keratoses and crusts on the top of a lesion, as described above, to help ultrasound penetration to the desired level in the skin.

## 4 DISCUSSION

Twenty megahertz HIFU treatment of AK in this introductory study had a very high cure rate. HIFU also had a favorable safety profile with no significant sequela observed, that is, no cases of scar nor dyspigmentation. The HIFU‐induced superficial dry ulceration or crustation healed within a few weeks, and no case of infection was noted. The treatment was painful; however, patients who had received PDT in the past unanimously reported HIFU to be less painful and more acceptable. Pain after HIFU is furthermore short and confined to the site of the ultrasound shot in contrast to pain after PDT and lasers, which is more protracted and may require treatment with cool‐packs, or pretreatment with local or systemic analgesics. This was not required using HIFU.

High‐intensity focused ultrasound is in comparison with cryotherapy with liquid nitrogen, commonly used in dermatological practice, more complicated to use and more resource‐demanding. Many patients only have few and sporadic AK responding well to cryotherapy, and some are treated with topicals.^20^

However, some patients with fair skin and years of sun exposure suffer multiple AKs and belong to a different category. AKs are premalignant and may develop into BCCs and SSCs, and many patients with multiple AKs have been subject to surgical treatments including Mohs surgery for complicated cancers.^21^ Predilection sites are the sun‐exposed sites, namely scalp, face, shoulders, upper trunk, forearm, and legs. PDT is a popular treatment of multiple AKs and positioned as a standard treatment of choice supported by a European guideline.^22^ However, PDT is resource‐demanding for the patients, who are off job for most of a day, and for the clinics and the health system. Furthermore, PDT using lamps is limited to the few anatomical sites, which can be dosed occlusively with the photosensitizer; the treatment is very painful. PDT has significant limitations in the field control of AK, and there is a need for an alternative.^23^

This exploratory study indicates that 20 MHz HIFU used for AK has important advantages over PDT practically, resource‐wise, with respect to efficacy, as well as with respect to pain during treatment. HIFU can be applied to multiple sites of the body in one brief session. Twenty megahertz HIFU therefore has the potential to replace PDT in future.

The study, supported by the literature about use of HIFU for internal cancers, furthermore indicates that 20 MHz HIFU can be used for different skin cancers, particularly BCC, different premalignant conditions, and for a range of benign tumors. Furthermore, difficult‐to‐treat Kaposi Sarcoma is a potential indication, supplementary to radiotherapy or as first‐line approach.

In such treatments of skin cancers, more experience is needed regarding high‐power treatment, “sandwich treatment,” and “two‐pass treatment,” where the same dose is applied once more to the same lesion immediately after the first dose, in order to raise the temperature in the target further and enforce the effect on the pathology. Application of HIFU to cancers is bound to be more expertise demanding and more dependent on the operator as compared with treatment of AK, which probably can be delegated to trained nurses.

There is a need for confirmative studies, and particularly a head‐to‐head comparison, between PDT and 20 MHz HIFU in treating AK.

## CONFLICT OF INTEREST

This study has been funded by TOOsonix A/S, Denmark.
